# Reproducibility of Cycling Kinetics on an Ergometer Designed to Quantify Asymmetry

**DOI:** 10.3390/s26010320

**Published:** 2026-01-03

**Authors:** Sierra Sweeney, Shahram Rasoulian, Atousa Parsaei, Hamidreza Heidary, Reza Ahmadi, Samira Fazeli Veisari, Saied Jalal Aboodarda, Amin Komeili

**Affiliations:** 1 Department of Biomedical Engineering, University of Calgary, Calgary, AB T2N 1N4, Canada; sierra.sweeney@ucalgary.ca (S.S.); shahram.rasoulian@ucalgary.ca (S.R.); atousa.parsaei@ucalgary.ca (A.P.); samira.fazeliveisari@ucalgary.ca (S.F.V.); 2 Department of Mechanical and Manufacturing Engineering, University of Calgary, Calgary, AB T2N 1N4, Canada; seyedhamidreza.heida@ucalgary.ca (H.H.); reza.ahmadi3@ucalgary.ca (R.A.); 3 Faculty of Kinesiology, University of Calgary, Calgary, AB T2N 1N4, Canada; saiedjalal.aboodarda@ucalgary.ca

**Keywords:** biomechanics, cycling kinetics, limb asymmetry, reliability

## Abstract

**Highlights:**

**What are the main findings?**

- This device consistently measured pedal force, power, handlebar force, and seat post mediolateral shift across both incremental and constant load protocols.
- Lower limb normalized symmetry index values were reproducible and not altered by fatigue in a task failure test.

**What are the implications of the main findings?**

- The ergometer can serve as a reliable tool for measuring lower limb asymmetry.
- The ergometer has the potential to be used in rehabilitation settings.

**Abstract:**

Cycling-based rehabilitation is a non-invasive intervention for individuals with lower limb asymmetries. However, current cycling devices lack comprehensive biomechanical feedback and cannot assess asymmetry. Our lab has developed a novel cycle ergometer equipped with three-dimensional force pedals, a seat post and handlebar force sensors, which allow for a comprehensive analysis of asymmetry across a fatiguing task. This study assessed the reproducibility of the cycling kinetics and asymmetry index derived from this device during incremental and constant load cycling tasks to volitional failure. Eighteen participants completed incremental and constant-load tests, each across two identical sessions. Pedal forces and power were analyzed for each leg individually, and handlebar forces and seat post mediolateral sway were recorded during cycling. Normalized symmetry index (NSI), a metric quantifying the degree of asymmetry between limbs, was calculated for each variable. The reproducibility of the device was assessed using repeated measures analysis of variance and intraclass correlation coefficients (ICC). No significant session or interaction effects were found for pedal, handlebar, and seat post measures (all *p* > 0.05). Time effects were observed for pedal force and power in the incremental test (all *p* < 0.001). NSI values were reproducible with high ICC values (≥0.70) for force and power. The results suggest that this ergometer offers reproducible cycling kinetics and asymmetry measures across a fatiguing task. The findings support the application of this ergometer in research and rehabilitation settings.

## 1. Introduction

Rehabilitation programs using cycling exercise are widely recognized as effective non-invasive interventions for various musculoskeletal conditions, such as knee osteoarthritis (KOA) [1], knee ligament injuries [2], and balance impairments [3]. Muscle weakness and joint pain, common symptoms of these conditions, often affect only one leg, leading to functional asymmetry between the limbs [4]. While a limb asymmetry level below 5% is considered normal, approximately 25% of the global population exhibits asymmetry greater than 15% and could benefit from cycling-based rehabilitation interventions [5,6]. Such asymmetry can lead to chronic anatomical and functional complications, including but not limited to an increased risk of falls and injury, low back pain, and further joint degeneration such as osteoarthritis [1]. Additionally, it has been shown that limb asymmetries can persist for six months postoperatively from anterior cruciate ligament reconstruction, highlighting a strong need for a device that can monitor this more closely [7,8]. These findings highlight a significant demand for innovative rehabilitation ergometers capable of providing real-time, bilateral kinetic and kinematic feedback during cycling. Such devices can play a crucial role in detecting asymmetry and optimizing individualized rehabilitation programs [9,10].

Prior investigators have attempted to design cycle ergometers equipped with pedal sensors to detect asymmetry during various cycling tasks. For instance, García-López et al. [11] used two-dimensional (2D) force analysis to identify asymmetry during a modified incremental task and self-selected power test in healthy individuals. However, their analysis focused solely on comparing right and left pedal torque and did not quantify asymmetry levels between limbs. In another study, Carpes et al. [12] used 2D force analysis to assess bilateral pedaling asymmetry during a simulated 40 km time trial, in which participants were instructed to complete the task as quickly as possible. However, the use of 2D force analysis in both studies limited their ability to assess asymmetries across all three force vector components. In addition, neither study normalized their force data prior to analyzing asymmetry, a crucial step to avoid skewed inter-participant comparisons. More recently, Martín-Sosa et al. [13] utilized three-dimensional (3D) force pedals, where the greatest asymmetry was found in the mediolateral force component. Although they normalized their data by calculating the symmetry rate between dominant and non-dominant legs at each time point, a notable limitation was that all participants cycled at a constant power output of 150 W, regardless of their peak power levels. This uniform workload could have induced varying levels of metabolic stress and neuromuscular fatigue across participants, potentially confounding the asymmetry results. In addition, the ergometers used in these investigations were equipped only with pedal force sensors and did not measure forces at the seat post or handlebars [11,14]. Therefore, developing a novel ergometer equipped with additional sensors is necessary to detect a broader range of asymmetry indices.

Bini et al. developed a custom Lode Excalibur ergometer equipped with Sensix force pedals to assess the reproducibility across several different power outputs and cadences [15]. The focus of this study was to compare the difference in power output between the pedals and the ergometer [15]. However, exercise tasks in this study were not continued until task failure. This protocol does not consider how fatigue impacts the reproducibility of force between sessions. Additionally, only instrumenting the ergometer with force pedals did not provide full insight into the biomechanical factors that may impact reproducibility.

To address these limitations, our laboratory has recently developed a custom cycle ergometer equipped with three-dimensional pedal force sensors, a seat post sensor, handlebar and pressure grip sensors. This system is designed to measure force, power, and torque outputs, along with their corresponding asymmetries between the lower limbs. It also captures mediolateral seat post forces and handlebar pressure, offering a more comprehensive analysis of cycling mechanics. Before using this device for rehabilitation purposes in clinical populations with lower limb asymmetry, it is essential to investigate the reproducibility of bilateral force and power as well as the potential asymmetry in these variables across cycling performed to task failure. Therefore, this study evaluated the reproducibility of key mechanical outputs (e.g., pedal force, normalized symmetry index, handlebar force, and seat post force) during maximal step incremental and constant load cycling protocols. We hypothesize that the newly developed ergometer will provide reliable measurements of bilateral asymmetry under incremental and constant-load fatiguing cycling tasks, thus representing a robust instrument for clinical and scientific applications.

## 2. Materials and Methods

### 2.1. Participants

Eighteen healthy individuals (11 females, 7 males; age: 22.55 ± 4.54 years; height: 171.42 ± 7.70 cm; body mass: 70.56 ± 12.53 kg) were recruited for the study. Inclusion criteria were age between 18 and 50 years and successful completion of the Physical Activity Readiness Questionnaire (PAR-Q+). Exclusion criteria included a body mass index (BMI) greater than 30 kg/m^2^, regular smoking or tobacco use within the past year, or any known neurological, cardiovascular, metabolic or other health conditions likely to interfere with strenuous exercise performance or confound the study results. Written informed consent was obtained from all participants prior to participation. Individuals were asked to refrain from vigorous physical activity for 24 h and from consuming alcohol or caffeine for 12 h before each testing session. Ethics approval was obtained from the University of Calgary Ethics Board (REB#24-1803).

### 2.2. Experimental Protocol

Participants completed one familiarization session and four experimental sessions. Prior to all testing sessions, the bike and sensors were calibrated. The force pedals were calibrated by performing two full rotations of the crank to ensure proper initialization. Following this, the left crank arm was positioned in a forward and horizontal orientation with both pedals completely unloaded to establish a zero-force reference. The handlebar and seat sensors were also unloaded during calibration to set their baseline values to zero. During the familiarization session, participants were introduced to the study procedure and cycling tasks. Saddle height and handlebar position were individually adjusted for each participant to ensure proper bike fit [16]. The next two sessions involved a repeated incremental cycling test. After a 3 min warm-up at 40 W and 70 rpm, participants completed the incremental test, during which power output increased by 20 W every minute. The test continued until volitional task failure or until the participant was unable to maintain the required power output for 10 s, despite verbal encouragement.

The final two sessions consisted of constant-power cycling trials. Following the same 3 min warm-up at 40 W and 70 rpm, participants cycled at a constant power set to 70% of their peak power output, as determined from the incremental test. In all testing sessions, participants were instructed to maintain a cadence of 70 rpm and continue exercising until they reached volitional task failure or could no longer sustain the required power output with a variation exceeding ±20% for 10 s. The 70% peak power level (mean ± SD: 162.81 ± 36.81 W) was chosen to delay the onset of fatigue, allowing sufficient time to analyze fatigue-related changes in kinetic symmetry between the lower limbs. To ensure consistent posture and minimize variability in force values, participants maintained seated cycling with hands on the handlebars throughout each trial. Power output and exercise duration were not visible to participants and were monitored in real time by the researchers. To ensure adequate recovery, a minimum of two full days of rest was provided between sessions.

### 2.3. Experimental Setup

#### 2.3.1. Custom Instrumented Cycle Ergometer

A custom, sensor-integrated Echelon EX-5 stationary bike (Echelon Fitness Multimedia LLC, Chattanooga, TN, USA) was developed to enable high-precision biomechanical data acquisition. The system captures real-time, 3D pedal forces, crank angle, saddle and handlebar forces and moments, and grip pressure, supporting detailed analysis of whole-body cycling mechanics.

#### 2.3.2. Pedal System

**Figure 1:**
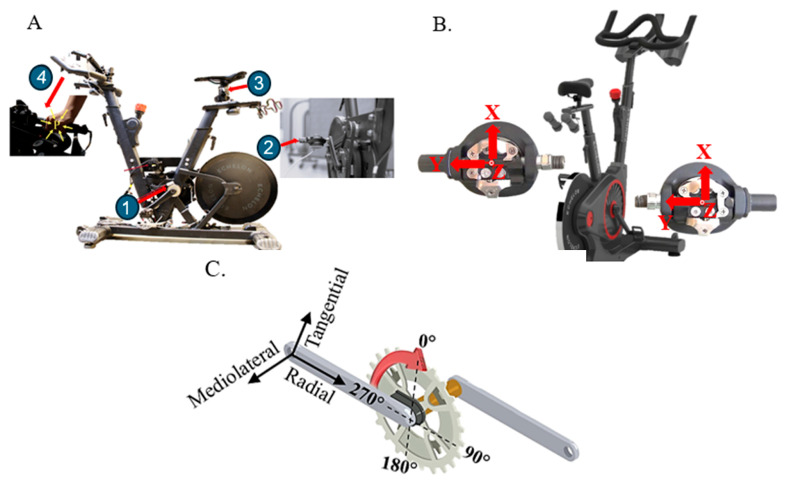
Panel (**A**) shows the cycling ergometer, consisting of (1) crank encoder, (2) force pedals, (3) saddle force sensor, and (4) handlebar sensors. Panel (**B**) demonstrates the three directions of pedal force components with respect to the pedals. Panel (**C**) illustrates the coordinate system with respect to the crank as well as the definition of the crank angle.

Each pedal integrates a Sensix ICS-MB six-axis force-torque sensor employing six strain gauges (Wheatstone bridges) to measure Fx, Fy, Fz, and Mx, My, Mz (Figure 1C). The pedal body (84 mm × 45.2 mm, 450 g) houses a 53 mm × 22.2 mm sensor core. The sensor has a specified natural frequency of 13 kHz and measuring ranges of ±1200 N (Fx, Fy), ±4700 N (Fz), and ±90/±40 Nm about the x- and y/z-axes, respectively (Mx, My/Mz), with overload tolerances up to 6470 N (force) and 230 Nm (moment).

Each pedal also includes a Scancon SCA16 encoder (D: 16 mm × H:20 mm, 5000 PPR; Scancon Encoders A/S, Hillerød, Denmark). Global crank angle is referenced using an encoder (RLS-MR076G060A120N00, RLS Merilna tehnika d.o.o., Komenda, Slovenia). Together, these encoders enable mapping of pedal-frame forces into a crank-based frame with tangential, $F_{T}$ (to the trajectory of crank end), radial, $F_{R}$ (following the direction of the crank axis), and mediolateral, $F_{ML}$ (direction of the pedal axis), components (Figure 1C).

#### 2.3.3. Seat Post Sensor

A Sensix I-Seatpost six-axis force-torque sensor (27 mm height, 53 mm diameter, 180 g; Sensix, Poitiers, France) captures saddle interaction forces and moments. The unit has a specified natural frequency of 13.3 kHz and measuring ranges of ±500 N (Fx, Fy), ±1200 N (Fz), ±150 Nm (Mx, My), and ±20 Nm (Mz), with overload capacity up to 6330 N (force) and 310 Nm (moment). The analog output is ±10 V per channel. The sensor is rated IP65 and operates from −20 to +70 °C.

#### 2.3.4. Handlebar Sensor System

The handlebars are equipped with Sensix I-Handlebar six-axis force-torque sensors (27 mm height, 53 mm diameter, 180 g; Sensix, Poitiers, France) and power grip manipulandum (PGM) grip-force sensors (120 × 32 mm, 96 g). The PGM sensors measure grip force over 0–1000 N with <0.1% of measurement range (MR) error, and their construction is designed to be insensitive to contact location, providing consistent output across the handle surface.

#### 2.3.5. Data Acquisition and Signal Conditioning

Two dedicated data-acquisition (DAQ) systems (Sensix, Poitiers, France) are used for signal collection and conditioning. Pedal signals are routed through a Sensix DAQ populated with six INA2128 instrumentation amplifiers (Texas Instruments, Dallas, TX, USA), using 4.5 V excitation (±0.002 V), a gain of 1370, and analog low-pass filtering at 2000 Hz. Saddle and handlebar sensors are connected to a separate DAQ unit with a similar conditioning architecture but configured with a gain of 912. The following variables were analyzed: pedal forces, power, cadence, saddle pressure, and grip force. The software stores all measured parameters as time-synchronized text files for offline analysis.

#### 2.3.6. Coordinate Mapping

Figure 1B illustrates the pedal coordinate system: Fx (perpendicular to the pedal axis and parallel to the pedal surface), Fy (along the pedal spindle/rotation axis), and Fz (normal to the pedal surface). Using the onboard encoders and the global crank angle reference, pedal-frame forces are transformed into crank-based and global coordinates for biomechanical interpretation.

### 2.4. Signal Processing

Variables of interest included minimum, maximum, average, and standard deviation of pedal forces, handlebar forces, and saddle forces. Five normalized time points were defined within each test: start (0%), early (25%), midpoint (50%), late (75%), and final (100%). In the incremental test, time points were mapped to power labels (incremental numbers) computed as a percentage of the maximum power label (Pmax) and rounded down to the nearest integer. For the constant-power test, time points corresponded to the same proportions of the total test duration. Therefore, the time intervals for each participant depended on their individual test duration. Each time point encompassed over a ±2% of the participant’s total test duration. Warm-up data were excluded from all analyses.

NSI was computed as [13]:

(1) $$\mathrm{NSI}(\%)=\left(\frac{\left(D-\mathrm{ND}\right)}{(D+\mathrm{ND})/2}\right)\times 100$$

where D denotes the dominant limb and ND the non-dominant limb. Leg dominance is frequently defined as the leg the participant prefers to kick a ball with [17]. In this study, leg dominance was determined by verbal confirmation for each participant. Melick et al. [18] reported 100% agreement between self-reported and the observed dominant leg, which supports its validity as a reliable measure of limb dominance. All participants were right leg dominant.

Whereas all participants were right leg dominant besides one, right leg was utilized for dominant limb in all calculations. The absolute value of NSI was analyzed to quantify the magnitude of inter-limb asymmetry.

### 2.5. Statistical Analysis

All statistical analyses were performed on the absolute values of each variable of interest using IBM SPSS, version 30 (SPSS Inc., Chicago, IL, USA). Shapiro–Wilk and Mauchly tests assessed the normality and sphericity, respectively. All variables met normality assumptions; when sphericity was violated, the Greenhouse-Geisser correction was applied and reported. Paired-samples *t*-tests compared (i) time to exhaustion in identical sessions and (ii) peak power output across the two incremental sessions. Two-way repeated measures ANOVAs were run separately for the incremental and constant-power tests with within-subject factors session (2 levels) × time (5 levels: 0%, 25%, 50%, 75%, and 100%). Statistical significance was set at *p* < 0.05. Intraclass correlation coefficients (ICCs) with 95% confidence intervals were calculated for both the incremental and constant-power tests using a two-way mixed-effects model in SPSS statistical package version 30 (SPSS Inc., Chicago, IL, USA). Descriptive statistics are reported as mean ± standard deviation.

## 3. Results

Maximum power outputs achieved in session 1 (228.40 ± 49.98 Watts) and session 2 (229.47 ± 50.83 Watts) of the incremental tests did not show a significant difference between the two trials (*p* = 0.835). Similarly, there was no statistical difference in time to task failure for session 1 (13.79 ± 5.02 min) and session 2 (15.67 ± 9.33 min) of the constant load cycling trials (*p* = 0.109).

**Figure 2:**
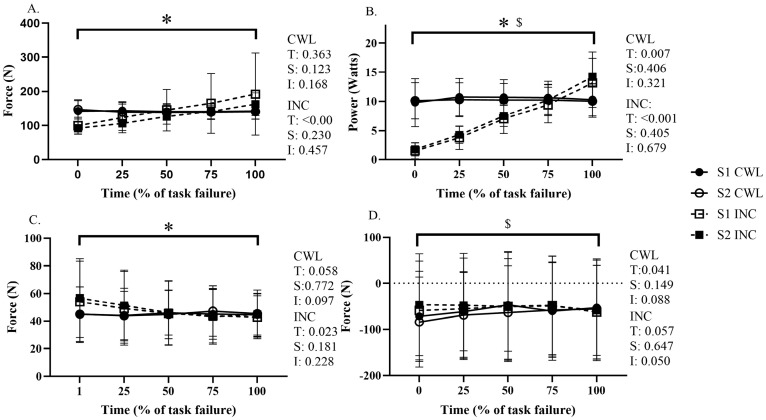
Right (**A**) pedal average force across all four sessions, (**B**) average power across all four sessions, (**C**) handlebar average force across all four sessions. (**D**) Mediolateral seat post shift across all four sessions. Constant workload (CWL) test, incremental (INC) test. Time (T), session (S), interaction (I). * Indicates a significant change across time for the incremental tests. $ Indicates significant change across time for the constant workload test. Dashed lines indicate the incremental tests and solid lines indicate the constant workload tests.

**Figure 3:**
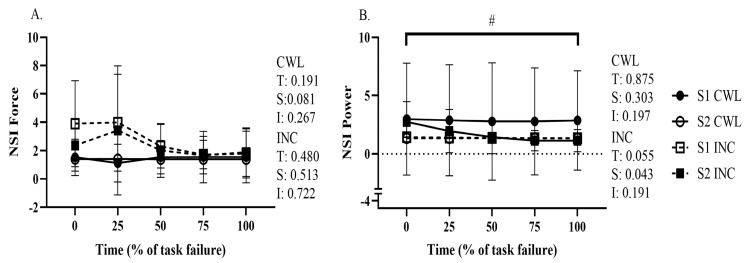
NSI (**A**) Force, (**B**) Power, across all sessions. # indicates a session effect for the incremental test. Dashed lines indicate the incremental tests and solid lines indicate the constant workload tests.

For the incremental trials, the values presented in Figure 2 (and Table A1 presented in Appendix A) indicate that there was no session (trial) or interaction effects for right pedal average force (*p* = 0.230, *p* = 0.457), right pedal average power (*p* = 0.405, *p* = 0.679), right handlebar average force (*p* = 0.181, *p* = 0.228), respectively. Significant time effects were observed for right pedal average force (*p* = <0.001), right pedal average power (*p* = <0.001) and right handlebar average force (*p* = 0.023). As shown in Table 1 and Figure 2, the device provided a reliable capture of pedal force and power as well as handlebar and seat post properties. All variables demonstrated good to excellent reproducibility. In addition, the stability of NSI across time and sessions (Figure 3) suggests that the ergometer can provide consistent measurements of inter-limb symmetry under fatiguing conditions, which is crucial for monitoring potential alteration in asymmetry induced by fatiguing tasks.

For the constant load tests presented in Figure 2 (and Table A2 presented in the Appendix A), no session or interaction effects were observed for right pedal average force (*p* = 0.123, *p* = 0.168), right pedal average power (*p* = 0.406, *p* = 0.321), right handlebar average force (*p* = 0.772, *p* = 0.097) or seat post mediolateral shift (*p* = 0.149, *p* = 0.088), respectively. There was no time effect for right pedal average force (*p* = 0.363) or right handlebar average force (*p* = 0.058) (Figure 2); however, a significant time effect was observed for right pedal average power (*p* = 0.007), left pedal minimum force (*p* = 0.012) and seat post mediolateral shift standard deviation (*p* = 0.006) values. Unlike the incremental test, most kinetic variables remained stable during the constant-load trials, illustrating the device’s ability to reliably capture steady-state mechanical responses over prolonged cycling.

ICC demonstrated excellent reliability between sessions for both the incremental test and the constant load test (Table 1). This indicates that the device has very strong test–retest reliability.

For the incremental test and constant load test, as depicted in Figure 3, no significant differences were found in NSI between sessions for force and power. The only exception was a session effect observed for power during the incremental task (*p* = 0.043). Indeed, the stability of NSI under fatiguing conditions is a notable strength of the device, indicating its ability to provide consistent and reliable measurements during physically demanding tasks. This reproducibility is particularly valuable for prescribing rehabilitation programs or performance monitoring, where subtle changes in asymmetry need to be detected over time.

ICC demonstrated excellent reliability among NSI between sessions for both incremental and constant load tasks (Table 2).

## 4. Discussion

The purpose of this study was to assess the repeatability of an instrumented cycle ergometer across incremental and constant power trials. The results suggest that the cycle ergometer was a reproducible device in its ability to measure pedal force, handlebar force and mediolateral sway in the seat post. In addition, the ergometer was a reproducible device in quantifying NSI between sessions in both exercise tasks. Overall, the ergometer provided reproducible quantification of lower-limb kinetics and asymmetry throughout fatiguing protocols.

The ANOVA analyses demonstrated no significant difference between exercise sessions and ICC exhibits excellent reproducibility across testing sessions and tasks for measures of total force and power for both right and left sides pedal sensors, handlebar sensors and seat post sway. As there was no significant effect observed between sessions in either the step test or constant load test, it can be concluded that even though the participants experienced exhaustion across the task, the instrumented cycle ergometer was still able to detect reproducible NSI values across the fatiguing tasks.

This observation was consistent for both the incremental and constant load tests. The reproducible time effect that was observed in the incremental tests for both force and power of the right pedal demonstrates that as resistance increased, so did the workload of the participant. As resistance increased during the incremental test, the participants must have expectedly exerted more force on the pedals to maintain the required power output. This expected time effect for the right and left pedal force, and power was accompanied by the excellent reproducibility of these measures between sessions. The reproducible time effects observed for pedal force and power during the incremental test align with expected neuromuscular adjustments to increased resistance. These findings confirm that the ergometer can accurately capture dynamic adaptations in lower-limb kinetics. By capturing both progressive adaptations during the incremental test and stable steady-state outputs during the constant-load protocol, the ergometer demonstrates its capacity to reliably characterize a wide spectrum of cycling behaviors. This dual capability is particularly valuable for evaluating both performance progression and fatigue-related stability. The stability of NSI under fatiguing conditions is a notable strength of the device, indicating its ability to provide consistent and reliable measurements during physically demanding tasks. This reproducibility is particularly valuable for longitudinal rehabilitation or performance monitoring, where subtle changes in asymmetry need to be detected over time. Similarly, the majority of the measures quantified during the constant load trials demonstrated excellent reproducibility during sessions and across the cycling tasks to exhaustion. However, significant session values were observed for some measures, including left pedal average power for the incremental task (Table A1) and left pedal minimum and standard deviation force during the constant load test (Table A2). One plausible explanation for these observations could be that most participants were right-leg dominant, which means they may have been weaker and less coordinated on their left leg. In line with this observation, a study by Farrell et al. (2021) found that as participants became fatigued, they relied more on their non-dominant limb to avoid tiring their dominant limb prematurely [19]. This means that participants may have adopted different strategies to complete the constant load cycling task across sessions, resulting in different minimum forces being exerted on the pedals. Alternatively, this may reflect a non-physiological observation, as some of the participants were not experienced in cycling and might have adopted different strategies to complete the task between sessions.

Lower limb NSI was found to be consistent, with excellent ICC values for total force and power across all sessions and tests. However, the significant session effect observed for NSI of force during the incremental task could be due to the inclusion of healthy participants in this study. It is known that healthy individuals typically have a 5–20% NSI on any given day [19]. This means that one day a participant could have a higher NSI value than another day, resulting in inconsistent values. Even so, fatigue did not influence the reproducibility of NSI, as there was no significant difference between exercise trials across time. This means that consistent NSI values were seen between sessions, although the participants experienced considerable fatigue during the cycling tasks. The stability of NSI under fatiguing conditions is a notable strength of the device, indicating its ability to provide consistent and reliable measurements during physically demanding tasks. This reproducibility is particularly valuable for longitudinal rehabilitation or performance monitoring, where subtle changes in asymmetry need to be detected over time.

This novel ergometer addresses several limitations that exist in current research or commercialized devices. The ability of this device to measure 3D pedal forces allows for analysis of asymmetry in all 3 force vectors, unlike previous studies, which only utilized 2D analysis [11,12]. Normalizing data was also a key factor in this study to prevent skewed inter-participant comparisons and provide more accurate results, something that was not considered in previous studies [11,12]. Additionally, because participants completed an incremental test to task failure, the constant power output task could be prescribed at a relative intensity (i.e., 70% of their maximum power output) for each individual. This allowed participants to cycle at a workload based off their own maximal aerobic capacity. This approach addressed a limitation of the previous studies whereby all participants cycle at the same power level of 150 W [13]. This avoided false interpretations due to the task being too challenging or too simple for participants. Another novelty of the current work was the ability of the ergometer to measure pedal forces, handlebar forces and seat post sway. Including all these sensors allows for a more comprehensive overview of the biomechanical factors that play a factor in cycling mechanics.

The study by Bini et al., did find the reliability of the Sensix force pedals to be reliable in measuring power output between sessions and showed no significant differences [15]. This agrees with our results as there were no large discrepancies between the sessions in this study. Although protocols in the study by Bini et al. were different than the present study, this signifies the significance of exploring the effect of fatigue in the reproducibility of pedal forces and power. Additionally, this study explores additional biomechanical factors such as handlebar and seat post force.

There are a couple of limitations to this study which should be mentioned. Although the incremental task was required to be performed first to determine the power level for the subsequent constant load tests, this could possibly introduce order effects in this study. Another limitation to note is that the population of participants consisted of healthy young adults. Another limitation to note is that the population of participants consisted of healthy young adults. However, it was imperative to demonstrate that the ergometer could produce reproducible results with healthy participants prior to utilizing clinical populations.

In addition, cadence was not strictly fixed at 70 rpm, and some fluctuations occurred, which may have influenced the force, power and NSI values. This may partly explain the inter-individual variability observed and suggest that future studies should better control or account for cadence.

This novel device has several practical implications for various neuromuscular and physiological complications. Clinically, it could be utilized for rehabilitation from any disease that may result in asymmetry, including but not limited to stroke, osteoarthritis or cerebral palsy. This device would enhance current rehabilitation programs by providing in-depth biomechanical information about the asymmetry in force and power applied in each pedal as well as handlebar and seat post on the patient to the provider. Additionally, this device could be utilized to monitor rehabilitation progress in those with lower limb injuries including but not limited to knee replacement, anterior cruciate ligament reconstruction, and ankle surgery. This device would aid in safer return to sport protocols and may also be utilized to detect muscle asymmetries prior to their occurrence, minimizing injuries in athletes as well as the general population.

## 5. Conclusions

The findings of this study confirm that the instrumented cycle ergometer is a highly reliable and reproducible device for assessing key biomechanical parameters during cycling. Specifically, the ergometer consistently measured pedal force, handlebar force, and mediolateral shift in the seat post across both incremental and constant load trials. Furthermore, it demonstrated reproducibility in quantifying lower limb asymmetry (NSI) between sessions for both exercise tasks. The cycle ergometer also proved to be a consistent and reliable tool for detecting lower limb kinetics and asymmetry measures during fatiguing tasks. These results highlight the instrumented cycle ergometer’s potential as a valuable device for precise and repeatable biomechanical assessments in cycling research and rehabilitation analyses.

## Figures and Tables

**Table 1:** ICC (95% CI) for INC and CWL.

Variable	ICC INC	ICC CWL
Total Force L Pedal Maximum	0.951 (0.896–0.983)	0.988 (0.976–0.995)
Power L Pedal Average	0.959 (0.914–0.986)	0.981 (0.964–0.992)
Power L Pedal Maximum	0.959 (0.913–0.986)	0.986 (0.973–0.994)
Power L Pedal Minimum	0.931 (0.854–0.977)	0.920 (0.848–0.966)
Power L Pedal St.Dev	0.963 (0.922–0.988)	0.986 (0.974–0.994)
Power R Pedal Average	0.886 (0.758–0.961)	0.984 (0.970–0.993)
Power R Pedal Maximum	0.932 (0.856–0.977)	0.981 (0.964–0.992)
Power R Pedal Minimum	0.877 (0.738–0.958)	0.981 (0.964–0.992)
Power R Pedal St.Dev	0.934 (0.859–0.978)	0.977 (0.957–0.990)
Total Force L Pedal Average	0.958 (0.910–0.986)	0.987 (0.975–0.994)
Total Force L Pedal Minimum	0.965 (0.926–0.988)	0.705 (0.441–0.876)
Total Force R Pedal Average	0.906 (0.800–0.968)	0.981 (0.964–0.992)
Total Force R Pedal Maximum	0.931 (0.853–0.977)	0.982 (0.966–0.993)
Total Force R Pedal Minimum	0.854 (0.689–0.951)	0.946 (0.898–0.977)
Total Force L Pedal St.Dev	0.957 (0.909–0.986)	0.986 (0.973–0.994)
Total Force R Pedal St.Dev	0.884 (0.754–0.961)	0.984 (0.969–0.993)
Total Force R Handlebar Minimum	0.970 (0.936–0.990)	0.983 (0.969–0.993)
Total Force R Handlebar Maximum	0.989 (0.976–0.996)	0.970 (0.943–0.987)
Total Force R Handlebar Average	0.990 (0.979–0.997)	0.974 (0.950–0.989)
Total Force R Handlebar St.Dev	0.891 (0.769–0.963)	0.962 (0.928–0.984)
Total Force L Handlebar Minimum	0.956 (0.906–0.985)	0.896 (0.804–0.956)
Total Force L Handlebar Maximum	0.943 (0.879–0.981)	0.910 (0.829–0.962)
Total Force L Handlebar Average	0.963 (0.921–0.987)	0.901 (0.812–0.958)
Total Force L Handlebar St.Dev	0.901 (0.789–0.966)	0.915 (0.838–0.964)
Force y Seat Post Maximum	0.986 (0.969–0.995)	0.984 (0.969–0.993)
Force y Seat Post Minimum	0.990 (0.979–0.997)	0.991 (0.983–0.996)
Force y Seat Post Average	0.993 (0.985–0.998)	0.994 (0.989–0.997)
Force y Seat Post St.Dev	0.918 (0.826–0.972)	0.914 (0.836–0.964)

**Table 2:** Reported ICC (95% CI) Results.

Variable	ICC	95% CI
Force INC	0.816	0.650–0.922
Power INC	0.705	0.373–0.900
Force CWL	0.796	0.567–0.931
Power CWL	0.822	0.776–0.950

## Data Availability

Data are unavailable due to privacy or ethical restrictions.

## Abbreviations

The following abbreviations are used in this manuscript:

NSI	Normalized Symmetry Index
DAQ	Data Acquisition
ICC	Intraclass Correlation Coefficient
KOA	Knee Osteoarthritis
2D	Two-dimensional
3D	Three-dimensional
PAR-Q+	Physical Readiness Questionnaire
BMI	Body Mass Index
CWL	Constant Workload
INC	Incremental
MR	Measurement Range
T	Time
S	Session
I	Interaction
L	Left
R	Right
St. Dev	Standard Deviation

## Appendix A

**Table A1:** Incremental Test Reported *p*-Values.

Variables	Session	0%	25%	50%	75%	100%	Session	Time	Interaction
Total Force L Pedal Average	1	102.15 ± 20.42	118.00 ± 22.62	134.25 ± 21.64	150.99 ± 22.57	165.19 ± 34.35	0.216	<0.001	0.218
	2	98.41 ± 22.59	113.67 ± 25.62	129.67 ± 21.94	144.11 ± 24.62	161.72 ± 33.86			
Total Force L Pedal Minimum	1	45.17 ± 13.57	50.28 ± 19.78	39.92 ± 13.40	36.90 ± 10.60	27.47 ± 13.40	0.078	<0.001	0.411
	2	41.58 ± 15.31	44.34 ± 22.09	37.29 ± 15.40	31.36 ± 14.11	25.99 ± 14.13			
Total Force L Pedal Maximum	1	170.45 ± 38.86	218.38 ± 44.07	279.45 ± 48.28	338.62 ± 70.21	395.38 ± 104.62	0.713	<0.001	0.595
	2	164.10 ± 34.20	218.38 ± 49.07	282.23 ± 46.12	336.09 ± 61.83	390.90 ± 96.84			
Total Force L Pedal St.Dev	1	42.27 ± 14.10	57.32 ± 17.43	82.27 ± 17.62	105.83 ± 26.97	131.95 ± 36.82	0.571	<0.001	0.303
	2	41.03 ± 13.10	59.48 ± 17.86	85.09 ± 16.52	107.64 ± 23.61	130.89 ± 35.38			
Total Force R Pedal Maximum	1	157.84 ± 23.86	200.59 ± 31.89	262.22 ± 36.30	314.00 ± 56.27	371.14 ± 85.99	0.134	<0.001	0.388
	2	162.38 ± 32.55	218.47 ± 52.41	279.58 ± 55.31	332.53 ± 81.91	398.24 ± 125.48			
Total Force R Pedal Minimum	1	37.26 ± 11.62	41.61 ± 8.60	41.02 ± 11.56	39.36 ± 8.98	29.50 ± 7.49	0.332	0.570	0.406
	2	45.41 ± 23.24	55.59 ± 49.15	59.91 ± 69.80	62.55 ± 94.95	66.37 ± 130.19			
Total Force R Pedal St. Dev	1	39.42 ± 6.28	53.33 ± 8.80	76.66 ± 10.80	97.35 ± 21.52	123.58 ± 32.53	0.671	<0.001	0.471
	2	37.56 ± 9.38	54.96 ± 10.79	76.37 ± 15.87	95.41 ± 20.18	117.70 ± 32.96			
Power L Pedal Average	1	13.93 ± 7.00	34.37 ± 14.40	60.97 ± 16.00	85.21 ± 24.09	104.56 ± 26.91	0.042	<0.001	0.595
	2	16.61 ± 7.92	37.58 ± 14.32	64.88 ± 17.68	88.53 ± 24.34	111.31 ± 28.07			
Power L Pedal Maximum	1	172.32 ± 32.73	240.74 ± 43.30	319.99 ± 59.34	393.83 ± 89.67	435.03 ± 105.59	0.824	<0.001	0.110
	2	170.75 ± 31.49	239.12 ± 47.94	317.77 ± 60.48	388.38 ± 80.82	441.47 ± 100.03			
Power L Pedal Minimum	1	−96.24 ± 23.83	−96.03 ± 34.92	−83.20 ± 29.46	−69.85 ± 21.22	−43.82 ± 19.12	0.138	0.057	0.479
	2	−88.62 ± 28.99	−85.36 ± 34.57	−70.29 ± 26.50	−57.62 ± 23.56	−39.05 ± 23.80			
Power L Pedal St.Dev	1	89.55 ± 17.26	111.80 ± 19.99	133.28 ± 24.03	154.48 ± 29.43	161.43 ± 35.66	0.256	<0.001	0.400
	2	86.77 ± 18.47	107.81 ± 22.89	128.65 ± 24.21	148.99 ± 28.27	161.97 ± 34.38			
Power R Pedal Maximum	1	156.85 ± 16.18	217.48 ± 30.72	296.33 ± 46.91	359.74 ± 68.84	409.21 ± 91.17	0.824	<0.001	0.110
	2	153.68 ± 29.74	224.25 ± 38.60	300.97 ± 49.82	369.10 ± 69.04	424.44 ± 97.08			
Power R Pedal Minimum	1	−84.94 ± 27.94	−81.55 ± 30.90	−74.98 ± 34.17	−60.28 ± 26.04	−35.30 ± 26.56	0.152	0.013	0.379
	2	−96.24 ± 27.15	−102.52 ± 43.97	−95.31 ± 59.67	−92.51 ± 82.57	−78.39 ± 108.48			
Power R Pedal St.Dev	1	80.56 ± 13.92	98.99 ± 18.36	122.50 ± 23.77	138.74 ± 25.79	150.52 ± 31.59	0.118	<0.001	0.338
	2	84.28 ± 17.33	110.37 ± 27.61	134.00 ± 36.86	155.11 ± 49.87	170.39 ± 61.60			
Total Force L Handlebar Average	1	45.94 ± 22.45	44.31 ± 19.20	43.47 ± 13.71	43.68 ± 9.10	44.33 ± 7.37	0.262	0.809	0.121
	2	44.28 ± 20.47	42.51 ± 18.42	42.79 ± 13.70	42.89 ± 9.77	42.62 ± 8.83			
Total Force L Handlebar Maximum	1	57.60 ± 24.63	58.27 ± 21.56	59.59 ± 19.67	66.24 ± 17.22	71.93 ± 13.85	0.063	0.048	0.763
	2	54.39 ± 21.13	52.78 ± 19.66	54.43 ± 16.53	60.34 ± 13.79	67.55 ± 17.61			
Total Force L Handlebar Minimum	1	34.78 ± 20.10	31.95 ± 17.66	30.48 ± 13.36	27.33 ± 10.35	21.06 ± 6.79	0.450	0.041	0.167
	2	34.11 ± 19.09	32.72 ± 17.64	31.84 ± 12.72	28.71 ± 10.01	23.29 ± 6.66			
Total Force L Handlebar St. Dev	1	7.31 ± 4.24	8.61 ± 5.24	9.62 ± 6.56	12.78 ± 6.77	16.84 ± 5.57	0.096	<0.001	0.604
	2	6.56 ± 2.78	6.60 ± 3.47	7.45 ± 4.25	10.59 ± 3.97	15.01 ± 6.65			
Total Force R Handlebar Maximum	1	68.98 ± 30.77	64.15 ± 27.88	60.64 ± 28.54	61.98 ± 25.81	70.10 ± 25.70	0.063	0.048	0.763
	2	65.09 ± 31.17	60.05 ± 28.16	57.05 ± 26.67	60.58 ± 24.47	66.63 ± 26.02			
Total Force R Handlebar Minimum	1	44.89 ± 27.57	38.10 ± 24.62	32.26 ± 21.15	25.92 ± 17.41	20.37 ± 9.52	0.069	0.008	0.914
	2	44.04 ± 27.60	39.39 ± 24.74	34.13 ± 21.28	29.37 ± 16.81	22.12 ± 11.91			
Total Force R Handlebar St.Dev	1	7.56 ± 3.86	8.47 ± 4.42	9.41 ± 5.96	11.87 ± 6.10	16.57 ± 9.46	0.096	<0.001	0.604
	2	6.62 ± 2.79	6.67 ± 2.81	7.68 ± 4.10	10.36 ± 5.23	15.21 ± 10.17			
Force y Seat Post Maximum	1	92.01 ± 54.40	107.52 ± 51.00	117.07 ± 62.05	102.12 ± 57.94	77.40 ± 62.93	0.649	0.094	0.492
	2	94.79 ± 65.07	102.25 ± 66.49	109.46 ± 66.78	86.52 ± 66.41	73.85 ± 88.82			
Force y Seat Post Minimum	1	135.04 ± 72.10	119.83 ± 76.37	121.06 ± 79.18	121.04 ± 89.66	166.37 ± 127.88	0.944	0.084	0.126
	2	117.23 ± 74.26	121.58 ± 72.71	123.81 ± 73.47	135.90 ± 71.30	162.32 ± 100.48			
Force y Seat Post St.Dev	1	29.40 ± 18.09	25.43 ± 18.84	26.24 ± 20.64	33.01 ± 24.25	60.16 ± 30.53	0.192	0.006	0.140
	2	26.97 ± 21.92	24.23 ± 22.29	19.97 ± 16.91	30.70 ± 24.59	49.51 ± 34.79			

Left (L), right (R), standard deviation (St.Dev).

**Table A2:** Constant Load Test Reported *p*-Values.

Variables	Session	0%	25%	50%	75%	100%	Session	Time	Interaction
Total Force L Pedal Average	1	146.10 ± 22.76	144.67 ± 22.83	142.27 ± 22.62	141.45 ± 21.98	142.42 ± 26.53	0.218	0.012	0.265
	2	153.64 ± 25.68	146.40 ± 25.23	142.70 ± 25.32	144.10 ± 24.96	143.79 ± 26.00			
Total Force L Pedal Minimum	1	101.56 ± 63.01	103.01 ± 62.07	109.15 ± 58.00	101.88 ± 50.86	105.02 ± 50.19	0.420	0.064	0.477
	2	96.23 ± 67.12	98.42 ± 68.03	105.09 ± 70.18	97.26 ± 64.42	110.02 ± 49.39			
Total Force L Pedal Maximum	1	315.58 ± 76.25	318.30 ± 55.50	312.09 ± 59.03	313.58 ± 61.33	317.01 ± 73.81	0.218	0.12	0.265
	2	345.00 ± 64.31	325.20 ± 63.42	319.46 ± 67.96	326.10 ± 71.25	328.71 ± 75.04			
Total Force L Pedal St.Dev	1	98.57 ± 31.60	102.45 ± 20.64	100.15 ± 22.47	102.83 ± 24.02	102.66 ± 27.38	0.029	0.658	0.089
	2	110.18 ± 21.92	105.42 ± 22.70	103.78 ± 24.14	105.44 ± 26.04	105.84 ± 25.01			
Total Force R Pedal Maximum	1	304.31 ± 78.95	312.96 ± 55.40	306.62 ± 58.42	309.92 ± 60.43	308.29 ± 68.80	0.787	0.712	0.229
	2	319.04 ± 61.78	306.21 ± 53.79	304.10 ± 60.77	309.87 ± 63.10	311.14 ± 67.34			
Total Force R Pedal Minimum	1	37.23 ± 15.48	33.76 ± 11.70	35.38 ± 10.11	34.50 ± 8.76	35.92 ± 10.44	0.251	0.271	0.780
	2	35.29 ± 10.10	31.87 ± 8.68	32.42 ± 8.88	33.61 ± 8.51	33.17 ± 10.22			
Total Force R Pedal St. Dev	1	93.48 ± 31.01	100.17 ± 20.73	97.93 ± 23.12	99.35 ± 24.04	97.96 ± 26.62	0.637	0.651	0.152
	2	100.93 ± 21.66	98.65 ± 23.30	98.012 ± 23.30	98.30 ± 24.08	98.95 ± 24.39			
Power L Pedal Average	1	83.93 ± 33.08	91.06 ± 19.36	89.37 ± 20.07	88.17 ± 20.77	88.01 ± 22.72	0.880	0.370	0.233
	2	89.44 ± 23.94	88.45 ± 23.87	88.40 ± 23.47	86.57 ± 23.80	85.63 ± 22.72			
Power L Pedal Maximum	1	358.51 ± 92.70	383.24 ± 67.97	371.20 ± 70.16	371.30 ± 72.49	368.44 ± 87.50	0.236	0.456	0.117
	2	392.08 ± 77.68	380.22 ± 74.73	371.96 ± 83.10	375.44 ± 83.99	376.36 ± 90.57			
Power L Pedal Minimum	1	−64.71 ± 28.54	−63.13 ± 17.17	−62.02 ± 17.71	−63.94 ± 15.52	−68.59 ± 18.43	0.676	0.609	0.623
	2	−68.59 ± 27.76	−63.13 ± 25.84	−63.36 ± 24.02	−66.23 ± 23.78	−65.82 ± 24.39			
Power L Pedal St.Dev	1	143.86 ± 26.41	149.90 ± 23.44	146.44 ± 25.07	146.25 ± 24.96	145.92 ± 31.56	0.131	0.276	0.137
	2	156.05 ± 28.84	151.15 ± 29.35	147.37 ± 31.51	149.36 ± 30.98	148.23 ± 32.49			
Power R Pedal Maximum	1	341.33 ± 92.75	364.96 ± 67.17	361.09 ± 72.88	364.83 ± 75.89	357.17 ± 81.98	0.840	0.335	0.238
	2	357.72 ± 68.12	354.76 ± 63.04	353.23 ± 70.98	358.28 ± 71.06	356.92 ± 78.31			
Power R Pedal Minimum	1	−70.95 ± 32.51	−64.98 ± 28.20	−63.89 ± 25.96	−64.36 ± 22.52	−68.24 ± 25.77	0.209	0.264	0.261
	2	−72.93 ± 33.05	−66.12 ± 28.62	−69.52 ± 25.32	−67.94 ± 25.89	−69.77 ± 25.10			
Power R Pedal St.Dev	1	138.49 ± 31.79	144.35 ± 25.22	141.35 ± 25.12	141.87 ± 26.12	140.28 ± 27.22	0.826	0.749	0.229
	2	144.55 ± 31.79	139.94 ± 22.87	139.00 ± 23.53	139.67 ± 22.93	140.09± 24.96			
Total Force L Handlebar Maximum	1	59.44 ± 31.89	58.61 ± 29.18	62.41 ± 28.11	63.76 ± 33.71	68.91 ± 31.93	0.797	0.05	0.335
	2	63.68 ± 14.24	67.25 ± 16.28	68.13 ± 15.61	74.16 ± 19.25	71.59 ± 16.59			
Total Force L Handlebar Minimum	1	25.92 ± 17.63	23.71 ± 14.35	23.71 ± 13.17	22.34 ± 13.19	22.65 ± 13.16	0.824	0.353	0.288
	2	31.63 ± 15.58	27.02 ± 11.32	27.03 ± 10.58	26.09 ± 10.72	29.71 ± 16.44			
Total Force L Handlebar St. Dev	1	10.91 ± 7.32	11.56 ± 7.65	12.62 ± 7.66	13.02 ± 9.10	14.35 ± 8.68	0.893	0.09	0.410
	2	10.97 ± 4.60	13.03 ± 6.20	13.31 ± 6.34	14.93 ± 6.21	14.51 ± 6.53			
Total Force R Handlebar Maximum	1	56.62 ± 34.93	57.73 ± 30.62	62.34 ± 30.22	60.03 ± 31.21	65.64 ± 29.40	0.878	0.014	0.073
	2	60.96 ± 22.42	60.72 ± 22.47	63.99 ± 21.89	69.41 ± 21.52	68.27 ± 22.65			
Total Force R Handlebar Minimum	1	25.87 ± 25.70	25.90 ± 22.03	25.72 ± 21.66	25.49 ± 21.56	25.26 ± 18.48	0.173	0.177	0.080
	2	32.27 ± 21.54	32.51 ± 21.97	31.24 ± 21.04	32.24 ± 20.28	30.72 ± 19.71			
Total Force R Handlebar St.Dev	1	10.33 ± 6.09	10.70 ± 5.41	12.18 ± 6.29	11.36 ± 6.042	13.35 ± 6.32	0.412	0.013	0.547
	2	9.54 ± 5.04	10.14 ± 5.04	11.94 ± 6.42	13.60 ± 6.42	10.04 ± 6.95			
Force y Seat Post Maximum	1	107.01 ± 65.08	84.20 ± 62.03	96.08 ± 78.79	90.57 ± 69.84	88.18 ± 75.02	0.087	0.336	0.628
	2	82.66 ± 58.65	73.56 ± 50.60	70.95 ± 61.24	66.36 ± 64.45	68.36 ± 84.30			
Force y Seat Post Minimum	1	122.27 ± 71.95	118.81 ± 62.04	115.83 ± 64.88	137.59 ± 65.12	132.36 ± 66.06	0.106	0.114	0.138
	2	140.92 ± 82.33	132.90 ± 72.76	140.03 ± 77.36	141.30 ± 74.03	144.47 ± 78.60			
Force y Seat Post St. Dev	1	20.67 ± 21.75	23.84 ± 20.72	27.96 ± 27.25	28.50 ± 22.36	30.93 ± 27.28	0.786	0.006	0.825
	2	20.51 ± 17.04	21.05 ± 17.04	25.76 ± 20.66	26.22 ± 22.01	29.70 ± 24.36			
